# Problem-Based Learning for Interprofessional Education: A Review of the Concept and Its Application in a Geriatric Team

**DOI:** 10.7759/cureus.63055

**Published:** 2024-06-24

**Authors:** Mohammed Azzahrani

**Affiliations:** 1 Family Medicine, Rosedale Medical Center, Toronto, CAN

**Keywords:** healthcare professional training, quality of patient care, collaborative practice, geriatrics, team collaboration, multidisciplinary approach, geriatric care, interprofessional education (ipe), pbl, problem-based learning (pbl)

## Abstract

The global population is aging, with those aged 65 years or over increasing in number and accounting for a growing share of the population. There are increasing demands for geriatric care which makes the development and delivery of effective geriatric team training a priority. Training in geriatrics is complex because of the multiplicity of medical, psychosocial, and functional issues in elderly individuals which need to be addressed by a multidisciplinary approach using interprofessional education (IPE). Problem-based learning, a student-centered educational model that brings several natural strengths to IPE, is a unique curriculum replacing the traditional lecture-based learning model. This model enhances physician competency after graduation, mainly in psychosocial and teamwork issues that are fundamentally essential for geriatrics. IPE has been shown to have a substantial positive impact on team collaboration, individual development, and healthcare improvement. In this paper, we summarize the current findings from recent studies on training professionals from different healthcare disciplines to deliver care for the elderly in collaborative practice. We also discuss if an interprofessional problem-based geriatric team program in geriatrics is a promising solution to enhance professional collaboration and quality of patient care.

## Introduction and background

Global populations are aging with huge increases in the number of old and very old persons aged over 65 years [1]. Statistics Canada’s 2016 census showed that almost one in six Canadians (16.9%), totaling a record 5.9 million Canadians, was ≥65 years of age compared to 5.8 million (16.6%) children aged <14 years. For the first time, those aged 65 years and older outnumbered children 0 to 14 years of age. According to population projections, the share of people over the age of 65 is expected to increase to 25% by 2051 [2]. This will provide both opportunities and challenges for the specialty of Geriatric Medicine in Canada and across the world. A longer lifespan would be a curse without rendering comprehensive primary and specialty care to this extremely aged population in whom quality of life, independence, and function are of crucial importance [3,4]. Efficient care requires knowledge and skills in the management of various diseases and specific complex medical and psychosocial conditions related to older patients. While the awareness of geriatric medicine, as a medical specialty for caring exclusively for older patients, has largely improved in recent years [5], geriatric team training is still a critical issue in healthcare due to the rapidly increasing aging population and decreasing geriatric professionals [6,7].

There is an essential need for further development of geriatrics competencies among all medical students, primary care physicians, and other healthcare professionals to meet the needs of an aging society [8,9] that demands highly effective curricula in geriatrics. Training in geriatrics is complex due to the multiplicity of medical, psychosocial, and functional issues in patients with advanced age which often require to be addressed by a multidisciplinary approach using an interprofessional education (IPE) and collaborative practice (IPCP) [6,10,11]. Problem-based learning (PBL) is a student-centered educational model that brings several natural strengths to IPE [12]. Since its inception at McMaster University as a unique curriculum replacing the traditional lecture-based learning model, PBL has expanded across the world as one of the most common curriculum elements in medical education [13]. This educational strategy enhances physician competency after graduation, mainly in psychosocial and teamwork issues that are fundamentally essential for geriatrics [14].

Although IPE for healthcare professionals in geriatric care is crucial for quality care in older patients, most healthcare professionals do not have this opportunity to learn from this learning experience [15]. In this literature review, we summarize the current findings from recent studies on training professionals from different healthcare disciplines to deliver care for the elderly in a so-called collaborative practice [16]. We also discuss if an IPE program in geriatrics using the PBL approach is an effective way to increase professional collaboration and quality of patient care.

## Review

Interprofessional education

Definitions

The term “discipline” is defined as a “subject that is taught” or a “field of study,” while, by definition, a “profession” is “a calling requiring specialized knowledge and often long and intensive academic preparation” [17]. There is a global trend toward using the suffix “-professional” in the IPE literature [17]. The term “professional” includes the healthcare workers who provide the clients (i.e., patients, their families, and the community) preventive, curative, and rehabilitative care. Teamwork, which is often referred to as “Interprofessional Practice” in health care, brings about the collaborative and comprehensive care expected by the clients/patients. It is established that difficulties in communication and collaboration can lead to team failure and negative patient outcomes [18]. Interprofessionality is a cohesive practice between professionals from different disciplines, essential for their joint ability to provide a comprehensive and well-integrated solution to a client’s health problem [19]. As elaborated by D'Amour and Oandasan [9], the concept of “Interprofessionality” is defined as follows: “The process by which professionals reflect on and develop ways of practicing that provides an integrated and cohesive answer to the needs of the client, family, and populations. Interprofessionality requires a paradigm shift since interprofessional practice has unique characteristics in terms of values, codes of conduct, and ways of working.”

There is a significant gap between health education and healthcare delivery. Considering uniprofessional education (professionals from the same discipline), multi-professional education (different professionals brought together to understand a particular problem or experience), and IPE, as a continuum of learning with others, the movement through this continuum is characterized by increasingly complex knowledge and appreciation of other professions [20]. To link IPE to Interprofessional Practice and to ensure best practices, it appears essential to develop innovative educational and practice models of team-based, collaborative care by both faculties and professionals. IPE aims to help professionals reap the benefits of interprofessional collaboration. IPE aims to prepare healthcare professionals with the knowledge, skills, and attitudes necessary for collaborative interprofessional practice.

According to a well-recognized definition from the Centre for the Advancement of Interprofessional Education (CAIPE), IPE occurs when “two or more professions learn about, from, and with each other to enable effective collaboration and improve health outcomes.” This definition is broadly used across the world; for example, the World Health Organization (WHO) Study Group on Interprofessional Education and Collaborative Practice uses the CAIPE definition and is committed to IPE as part of the solution to mitigating “the global health workforce crisis” [21]. The WHO has published a framework for action including the key messages that: “IPE is a necessary step in preparing a ‘collaborative practice-ready’ health workforce that is better prepared to respond to local health needs. A collaborative practice-ready health worker is someone who has learned how to work in an interprofessional team and is competent to do so. Collaborative practice happens when multiple health workers from different professional backgrounds work together with patients, families, carers, and communities to deliver the highest quality of care. It allows health workers to engage any individual whose skills can help achieve local health goals” [21]. It is equally important to know what is not considered IPE; for example, if there is a lack of sharing of decision-making or responsibility for patient care or a lack of interaction in an interprofessional manner of care [22].

The Learning Process Involving Two or More Professions

IPE is essential for training the future workforce in healthcare. There has been a growing need for competency in working successfully with other healthcare professions as a major curricular requirement for future geriatricians. Primary care providers have also allied with geriatric specialists and other healthcare professionals about areas of mutual concern such as dementia, falls, and mental health. The lack of specialized preparation in these areas among primary care providers indicates an immediate need for evidence-based interprofessional curricular design and clinical training for geriatric concerns. Within an interprofessional group with different perceptions and expectations of learning progress, not only each member but also the group is responsible for the management of learning. This can be an early exercise of collaborative practice. All participants negotiate how, within the given objectives, to contribute to a process of cooperative, collaborative, reflective, and socially constructed learning [23,24]. For its success, the interprofessional learning process rests on the inclination and ability of learners to engage in new experiences, reflect on them from various viewpoints, generate hypotheses integrating their observations into logical theories, and apply these theories for making decisions and solving problems [25,26].

The interprofessional learning process is complex wherein the learning happens in “the zone of complexity” between familiar and unfamiliar tasks, and familiar and unfamiliar environments [27]. As participants individually, in pairs, or in groups assess their concepts, assumptions, and hypotheses, they reflect while identifying and acknowledging the uncertainty that arises [28]. The learning process is not limited to reflection-in-action (i.e., to thinking about or reflecting while you are performing the activity); it entails reflection-on-action (i.e., thinking about or reflecting about the practice undertaken after the event and turning that information into knowledge) [29]. It also goes beyond first-order reflection, in which participants are limited to their own professional and personal viewpoints, to second-order reflection, wherein they “de-center” their learning and consider points of view other than their own [30]. With interprofessional learning, there is some evidence of benefits to students, particularly in the form of improved skills of independent learning and better management of their cognitive dissonance [31]. Implicit behaviors and motives that affect attitudes and interprofessional relationships are also revealed by the process [32].

Approaches to Interprofessional Education

There are several equally effective approaches to IPE [33] that teachers can select from based on the particular needs of their learners.

Problem-based learning: This interprofessional learning method has been adapted into IPE from the medical field of professional education. It is now firmly established in medical schools and many educators consider it as the first choice if not as the only method [34]. PBL is especially suitable for IPE, as it is designed for participants to collaborate to critically evaluate and resolve the problem at hand. Hence, it encourages teamwork, strong integration of knowledge, and deeper learning which was commenced by WHO as the preferred learning method for interprofessional education.

Exchange-based learning: This approach, especially effective for teaching through case studies, encourages participants to share their feelings, compare perspectives, and exchange experiences with one another.

Practice-based learning: Interprofessional practice-based learning takes many forms, e.g., outplacement in another professional setting and linked learning for students concurrently on placement in adjoining workplaces.

Simulation-based learning: Role play allows participants to assume the perspectives of clients or other healthcare professionals. To give an example of a simulated interprofessional learning experience, a simulated ward was created for nursing and medical students at Dundee University, Scotland in early 1999 to train them in interprofessional collaboration in a realistic environment [35].

Observation-based learning: Several methods of observation-based learning exist. The traditional one consisted of job shadowing. More sophisticated methods, derived from a psychodynamic theory, were employed in training for psychotherapists. For instance, Guest et al. [36] developed an interprofessional practice placement for senior nursing students and junior medical students to provide them with an opportunity to learn together within an acute pediatric setting. The findings from the placement evaluation indicated that all participants valued their interprofessional learning experience and felt that there were improvements in their knowledge of one another’s roles, responsibilities, and clinical skills.

Psychodynamic theory methods were applied to train psychotherapists. Researchers developed IPE practice for nursing and medical students letting them work and learn together in the acute pediatric unit. The findings from these experience evaluations showed that students appreciated their IPE experience and acknowledged professional improvement in their skills.

E-learning (computer-based training): Advances in educational technology have had a great impact on the development of IPE. Telemedicine video conferencing technology, for instance, helps learners to interact with the teacher via online learning environments.

Received learning: Received or didactic learning, e.g., lectures, is the traditional pedagogy of a teacher-centered teaching strategy wherein the educator functions primarily as an authoritative figure, or, to a lesser degree, as both a guide and a resource for students. Didactic teaching has its role to provide structured inputs and systematic knowledge, but received learning occasionally seems necessary for complementing and reinforcing interactive learning [33].

Evaluation of Interprofessional Education

Evaluation of IPE is needed to produce evidence of success, measure it against specific learning outcomes, and identify and remedy any problems that may have occurred during the design or delivery of the program, all thereby strengthening the program moving forward. Freeth et al. [37] have developed a modified form of Kirkpatrick typology for evaluating IPE, as shown in Table 1. The outcome levels are not hierarchical and each may be sought for any IPE initiative.

**Table 1:** Modified Kirkpatrick’s model of educational outcomes for interprofessional education. From Freeth et al. (2002, p. 14) [37].

Educational outcome	Description
Reaction	Learners’ views on the learning experience and its interprofessional nature
Modification of attitudes/Perceptions	Changes in reciprocal attitudes or perceptions between participant groups; changes in perception or attitude toward the value; and/or use of team approaches to caring for a specific client group
Acquisition of knowledge/Skills	Including the knowledge and skills linked to interprofessional collaboration
Behavioral change	Identifies individuals’ transfer of interprofessional learning to their practice setting and changed professional practice
Change in organizational practice	Wider changes in the organization and delivery of care
Benefits to patients/Clients	Improvements in the health or well-being of patients/clients

Problem-based learning

PBL is a constructivist educational approach in which learners learn science and, as noted by Hmelo-Silver [38], develop critical thinking skills by attempting to find a solution to an open-ended problem. The use of small-group PBL sessions has been shown to be highly effective in developing problem-solving skills and teamwork [39]. Using a broad range of IPE models, it has been suggested that students require help from other health professions to solve frequently encountered problems that they could not solve on their own [40,41]. This interprofessional reliance starts to shape the attitudes and behaviors of students and leads to an appreciation of the interprofessional collaboration needed to optimally approach a patient or clinical problem [41].

History and Development of Problem-Based Learning

PBL may look like a 21st-century idea, but it is built on a delicate concept dating back to Socrates in the 5th century BCE. Socrates utilized problems with his students to help them explore how to learn through questioning, inquiry, and critical thinking [42,43]; all strategies that remain very relevant in today’s PBL groups. This concept continued throughout history; 20th-century American educational theorist and philosopher, John Dewey, challenged the traditional view of the instructor as the transmitter of static information and the learner as a passive recipient of knowledge [44]. He proposed preparing students for ongoing learning about a dynamic world as he mentioned “Education is not preparation for life; education is life itself.”

In the late 1960s, the Faculty of Health Sciences at McMaster University established a new medical school with an innovative educational approach that is now known across the world as PBL [45]. As stated by Bill Spaulding [46], a member of the original group of originators and later its historian, the idea of supplanting the traditional lecture-based learning model with the use of real-life clinical problems as a new learning model emerged following the observation that medical education did not become exciting for students until residency training when they were working with patients trying to save their problems. The initially enthusiastic medical students became disenchanted and bored with large amounts of information with little relevance to their future practice of medicine that they had to absorb as passive recipients [45].

PBL was not innovated out of the blue and the pioneers were influenced heavily by the Case Study Method pioneered at Harvard University in the 1930s [46]. Although the idea of using problems in education was not novel, what made the McMaster educational innovation unique was “the timing: students acquire the knowledge needed to solve problems after they began working on the problem situations” [46]. The original McMaster curriculum, throughout its entire three years, placed emphasis on small-group tutorials, life-long and self-directed learning, and a minimal number of didactic presentations, as well as the use of varied resources in learning, and the integration of biomedical and clinical sciences in the curriculum [13]. The first paper that describes the philosophy of the McMaster approach was published in 1974 by Neufeld and Barrows who both joined some years after the establishment of the program but soon played major roles in its further development [47]. Within a few years of its conception, PBL disseminated fairly fast into the curricula of medical schools around the world [48]. PBL has also expanded into a host of other disciplines beyond medical education, including other health sciences, social sciences, business education, veterinary medicine, forestry, and engineering [45].

The Process of Problem-Based Learning

In this student-centered educational model, as its name implies, learning is based upon a patient problem (trigger) as the mainstay of stimulating and structuring relevant learning. It has been defined as “both a method and philosophy involving problem-first learning via work in small groups and independent study” [14,49]. In the PBL process, students use “triggers” from the problem case or scenario to define their own learning objectives [50]. The students are enabled to understand the relevance of underlying scientific knowledge with principles in clinical practice while clinical materials are presented as the “trigger.” While solving the learning task (i.e., trigger), students work in a team called “tutorial groups” guided by tutors, who are facilitators, not teachers. The students discover new knowledge on their own with tutors’ guidance through brainstorming the problem and identifying what they need to learn from the trigger. Then, the members of the tutorial group share information and suggest different solutions.

The PBL process was pioneered by Barrows and Tamblyn in the medical school program at McMaster University in Hamilton, Canada [45]. Howard Barrows was a neurologist and already a well-known educator when he arrived at McMaster in 1971. In 1985, he described the basic outline of the PBL process as encountering the problem first; problem-solving with clinical reasoning skills and identifying learning needs in an interactive process; self-study; applying newly gained knowledge to the problem; and summarizing what has been learned [51]. In more recent years, the Maastricht “seven jump” approach (Table 2) [50] has become popular because it is designed as a “how to” list of instructions for the tutor and participant students in a PBL session. There are many derivatives of these two approaches; however, all of those are affected by and interested in the principle that learning is based upon a patient problem.

**Table 2:** The Maastricht “seven jump” approach for problem-based learning sessions.

The Maastricht “seven jump” approach	
Steps	Description
Step 1	Identify and clarify unfamiliar terms presented in the scenario; the scribe lists those that remain unexplained
Step 2	Define the problem or problems to be discussed; students may have different views on the issues, but all should be considered; the scribe records a list of agreed problems.
Step 3	“Brainstorming” session to discuss the problem(s), suggesting explanations based on prior knowledge; students draw on each other’s knowledge and identify areas of incomplete knowledge; the scribe records all discussion
Step 4	Review steps 2 and 3 and arrange explanations into tentative solutions; the scribe organizes the explanations and restructures if necessary
Step 5	Formulate learning objectives; the group reaches a consensus on the objectives; the tutor ensures that objectives are focused, achievable, comprehensive, and appropriate
Step 6	Private study (students gather information related to each objective)
Step 7	The group shares the results of the private study; the tutor checks the learning and may assess the group

The Effectiveness of Problem-Based Learning

Due to the worldwide spread of PBL in medical education over the past four decades, we are now in a position to consider its effectiveness compared to other educational strategies. The question is to what extent it prepares learners to be self-directed and life-long earners with a deeper knowledge of their discipline who are better prepared to use medical practice for patient care; this is what it was designed to do. There is a body of evidence supporting the superiority of PBL over the didactic teacher-centered approaches for developing competencies such as self-directed and life-long learning, effective interpersonal communication, and planning, as well as problem-solving, time management, critical thinking, and reflective evaluation among learners that are key desirable outcomes for healthcare professionals [52-55]. However, no consistent evidence has been provided suggesting PBL is superior to other educational strategies in improving students’ in-depth basic scientific and clinical skills, particularly as measured by performance in factual recall examination evaluations [52,56-66]. In one study on undergraduate psychiatry students, the PBL curriculum produced stronger academic performance in comparison with the traditional psychiatry curriculum [65]. Students who used strategic learning were female, had received the PBL curriculum, and were significantly more successful in both multiple-choice questions and the viva; between the curricula, no differences were noted in learning styles or attitudes to psychiatry [66]. In a recent randomized controlled study, the effects of PBL on knowledge acquisition (i.e., immediate post-test) and retention (i.e., delayed post-test after one week) were studied [66]. The study participants were randomly assigned to one of the three groups (i.e., PBL, lecture-based, and a self-study control group). Although no differences in knowledge acquisition and retention emerged between PBL and self-study groups, compared to students in a lecture-based method, PBL students performed better on a knowledge acquisition test but not on a knowledge retention test [67]. There have been other concerns about PBL as a learning approach. Albanese and Mitchell [62], for example, argued that it is a challenge to have outcomes that measure the actual success of PBL, that PBL graduates showed potentially essential gaps in their cognitive knowledge base, did not demonstrate expert reasoning patterns, and that PBL was costly. Vernon and Blake also noted it is very difficult to conduct high-quality comparative research on PBL that can be generalized owing to variations in definitions and implementations of PBL, conceptual problems in choosing and defining outcome variables, and the difficulty in study designs because of a wide range of methodological problems such as confounding, sampling bias, and failure to blinding [56].

As Strobel and van Barneveld suggested PBL can be a superior strategy to “train competent and skilled practitioners and to promote long-term retention of knowledge and skills acquired during the learning experience” [68]. Notably, the evidence suggests that PBL works better in particular medical contexts and in particular branches of medicine [54]. Below we will further discuss whether or not the advantages outweigh the disadvantages of applying interprofessional PBL as an educational module in geriatric medicine.

Application of Interprofessional Problem-Based Learning in Geriatric Medicine

Although older adults (>65 years of age) these days live longer and have less chronic disability than in the past, many still experience one or more geriatric syndromes (e.g., falls, delirium, and malnutrition) and are also prone to experience more mental health conditions (e.g., depression and anxiety). The current workforce is not large enough to meet the need for healthcare services by this age group at least partly because the caregivers are inadequately educated and prepared in geriatrics. The Institute of Medicine (United States) has identified a critical need for expanding geriatrics competence among all professional healthcare workforce, including all physicians, pharmacists, registered nurses, nursing assistants, and social workers [69]. The Partnership for Health in Aging states that most healthcare professionals “have not had sufficient opportunities to learn with, from, and about other healthcare professionals,” though it is a necessity for quality care for older adults [15].

In addition to managing geriatric conditions, many different types of healthcare professionals work together, including physicians, physician assistants, nurse practitioners, nurses, social workers, physical and occupational therapists, nutritionists, psychologists, pharmacists, and dentists. For example, an old adult with a urinary tract infection (UTI) may present with falling, confusion, agitation, dizziness, hallucinations, or other atypical symptoms without typical symptoms of dysuria, frequency, hematuria, lower abdominal pain, and fever. If UTI, as the treatable underlying etiology, is not found, it would be a wrong approach to treat only the symptoms overlooking the underlying cause. In other words, as geriatric patients may present with atypical symptoms, basic medical, psychosocial, environmental, and safety concerns should be screened by different healthcare professionals. Several studies have demonstrated that an interprofessional approach can decrease the occurrence and severity of geriatric syndromes such as delirium and falls in advanced age groups with higher morbidity and mortality [70].

The growing workload and the treatment complexity of older patients will need improved collaboration between the healthcare professions. This collaboration can be effectively achieved by interprofessional PBL which has been shown to contribute to patient safety improvement and in reduction of treatment errors [41,71-73]. Indeed, PBL represents a fruitful learning approach for paving the way for IPE [12]; the combination of IPE and PBL improves the attitudes of the healthcare professions toward each other [74] and has a positive effect on mutual respect and trust [73,75], enabling the group members to work and solve problems as a team [17,73,75,76]. Therefore, IPE activities most often use the classic model of PBL [77].

Despite this, to date, there is little data regarding the implementation of interprofessional PBL in the training of students and professionals serving geriatric clients and its impacts on the participating groups. Geriatric IPE programs have covered limited professionals such as general practitioners and practice nurses [78] or have been customized for a particular geriatric syndrome such as falls [79] or dementia [80]. An exemplary implementation of interprofessional PBL for undergraduate students in Occupational Therapy and Physiotherapy to understand their various roles on interprofessional healthcare teams is reported by Richardson et al. at McMaster University [81]. The authors found that students who learned together in interprofessional tutorial groups using healthcare problems related to geriatric clients developed skills necessary to function on interprofessional teams, including team building, communication skills, and appreciation of the roles of others [81]. This may help students to develop a client-centered approach and prepare them for clinical practice on an interprofessional team serving geriatric clients, a population often with multiple health problems who require attention from professionals in different fields.

Another study was conducted in the School of Medical Sciences, Universiti Sains Malaysia (USM) wherein the main teaching-learning strategy in Phase II (Years 2 and 3) of the undergraduate course is PBL; all PBL problems used in Phase II of USM PBL curriculum of five academic sessions (1998-2003), in which age and presenting illness were mentioned, were analyzed [82]. The findings showed that problems of the PBL segment and examination questions were mostly designed to focus exclusively on young patients with rapidly resolving acute diseases. Only 17% of problems and 13% of examination questions included elderly people (aged >60), mostly in the early elderly (aged 60-74), with chronic, irreversible diseases; only one problem and one question featured late elderly (aged >70). This problem is not unique to USM, Malaysia. A review of the first and second-year cases (2004/2005) for age and issues relevant to geriatric medicine at Dalhousie University, Canada revealed that of 69 cases of the PBL curriculum, only 5 (7%) were 65 or older, and none were over 70 [83], and analysis of 162 PBL cases at two Australian medical schools showed only 4% of case patients were over 70 [84]. Such focus may contribute to the development of negative attitudes among the students toward the elderly and chronically ill patients [82,85].

In a recent study, 202 second-year medical students participated in a week-long PBL curriculum in geriatrics over two consecutive years that was found to be effective in promoting learning geriatric competencies [8]. Even though it was not an IPE program, medical students obtained in-depth learning across multiple geriatric competencies through contact with real cases in a PBL format. Due to timing constraints, the investigators only focused on core issues in the care of older adults, including falls, cognitive impairment, and caregiver stress. Polypharmacy and cognitive impairment were not as frequent as falls addressed by the students. This under-performance in these two areas was found in both years and the authors suggested a need for more emphasis on domains of medications and cognitive impairment in the curriculum [8].

Discussion

There is a substantial body of literature showing IPE may be useful for healthcare professionals to effectively work together and promote interprofessional competencies, including enhanced patient/client-centered care, communication skills, and professionalism. The enhancement of these competencies in professionals will lead to an improvement in the quality of health care and better patient outcomes [86,87]. The question is how it should be organized to achieve a desired result, particularly in the management of complex conditions. Health professionals can work together more effectively if they have been trained to do so by using IPE in their undergraduate or pre-licensure professional training programs. Theorists believe that for an effective undergraduate IPE, relevant, well-structured, realistic, and progressively more complex cases should be extensively used via the application of cooperative and experiential learning principles [76]. Johnson et al. [88] have described the five features of cooperative learning as follows: positive interdependence, face-to-face interaction, social skills, group processing, and individual accountability which must be included in every organized interprofessional study and case discussion [76]. Moreover, learners should cycle through the following four stages of experiential learning: planning, acting, observing, and, especially, reflecting [89]. PBL constitutes many of the essential principles of both cooperative and experiential learning and therefore brings several natural strengths to IPE [12]. Compared to case-based learning, a prominent strength of interprofessional PBL is the ability to incorporate multiple curricula with relative ease. Students are not necessarily prepared for the concepts and the problematic aspects of the PBL case before entering the process because they may learn from their own explorations between sessions and from each other [90]. An important feature of IPE is that the curriculum instead of having a focus on profession-specific skills should emphasize problems; it means an increased focus on the requirement for team collaboration. Therefore, gerontological and geriatric problems that highly need interprofessional engagement can be resolved more effectively [81,91]. The nationwide shortage of geriatric specialists indicates an immediate need for evidence-based interprofessional curricular design and clinical training in aging, dementia, and mental health, especially for primary care providers who lack specialized preparation in these areas [92]. Such educational activities should be reflected in “interprofessional collaborative practice” where they have the potential to affect the culture of collaborative practice and result in better patient and population health outcomes [93]. A “collaborative practice-ready” healthcare workforce that is competent in geriatrics principles can promote optimal aging and optimally manage multiple chronic conditions in advanced-age individuals [92]. The inclusion of issues relevant to aging and geriatric in the medical curriculum has been strongly advocated by WHO [94].

Examples of an interprofessional geriatric team training model curriculum and suggested geriatrics topics are presented in Table 3. A team of healthcare professionals will be formed according to the needs of geriatrics. The team will assign a tutor or a facilitator, not necessarily an expert in geriatrics, to prevent and manage any attitudes of superiority or inferiority among participants.

**Table 3:** Interprofessional geriatric team training model curriculum and suggested topics.

Interprofessional geriatric team training model curriculum and suggested topics	
Interprofessional geriatric team training model curriculum	Participants: Will include healthcare professionals and non-experts in the delivery of geriatric care, e.g., nurses, physiotherapists, occupational therapists, physicians, and pharmacists
	Facilitator/Tutor: Should be a non-expert to monitor social equality between participants
	Timing: A suggested schedule of timetable can be created to include the duration of meetings and assigned reading assignments
	Method: Will be problem-based learning in small groups
	Objectives: To improve geriatric care service and to enhance interprofessional team collaboration and individual development
Suggested curriculum topics for interprofessional education geriatric team training	Physiological changes with aging
	Falls in elderly
	Polypharmacy
	Dementia
	Deconditioning/Musculoskeletal disorders
	Depression in the elderly
	Urinary incontinence
	Pain management
	Chronic neurological disorders
	Parkinson’s disease

## Conclusions

No single healthcare professional could possibly become proficient in the wide range of knowledge and skills required for providing high-quality care for the elderly. An IPE program in geriatrics using the PBL approach is an effective way to increase professional collaboration and quality of patient care. Evidence suggests that PBL works effectively as an educational module in geriatric medicine. The inclusion of issues relevant to aging and geriatrics in the medical curriculum has been strongly advocated by WHO. Curriculum planners should regularly determine if these issues are adequately covered in the PBL curriculum, given the demographic trend toward an aging population. Emphasis given to such content may contribute to the development of knowledge and positive attitudes among the students toward older patients and individuals with chronic diseases and encourage students for the study of aging and careers that focus on the elderly and people who are chronically ill. It has been also shown that, when educated together in geriatric teams, different health professionals improve in attitudes toward teams and team skills. To prepare health professionals from other non-physician disciplines, formal curricula of interprofessional team training in geriatrics are required. This highlights the importance of IPE and collaborative practice between healthcare providers to promote health and provide care for older adults. Over the last several decades, substantial investments have been made in promoting IPE. However, much work remains to integrate IPE within clinical and research activities related to care for older adults.
